# Supplementary material for: Mechanistic origin of partial agonism of tetrahydrocannabinol for cannabinoid receptors
Source: J Biol Chem. 2022 Feb 26;298(4):101764. doi: 10.1016/j.jbc.2022.101764 (PMC8965160; doi:10.1016/j.jbc.2022.101764)
Supplement: Supplemental Figures S1–S22 and Tables S1 and S2 [file mmc1.pdf]

Supporting information: Mechanistic Origin of Partial Agonism of Tetrahydrocannabinol for Cannabinoid Receptors

Soumajit Dutta,[†] Balaji Selvam,[†] Aditi Das,[‡,¶,§,‖,⊥] and Diwakar Shukla[*,†,‖,⊥,#,@,△]

†*Department of Chemical and Biomolecular Engineering, University of Illinois at Urbana-Champaign, Urbana, IL, 61801*

‡*Department of Comparative Biosciences,University of Illinois at Urbana-Champaign, Urbana, IL, 61802*

¶*Department of Bioengineering,University of Illinois at Urbana-Champaign, Urbana, IL, 61801*

§*Department of Biochemistry,University of Illinois at Urbana-Champaign, Urbana, IL, 61801*

‖*Center for Biophysics and Quantitative Biology, University of Illinois at Urbana-Champaign, Urbana, IL, 61801*

⊥*Cancer Center at Illinois, University of Illinois at Urbana-Champaign, Urbana, IL, 61801*

#*National Center for Supercomputing Applications, University of Illinois, Urbana, IL, 61801*

@*Beckman Institute for Advanced Science and Technology, University of Illinois at Urbana-Champaign, Urbana, IL, 61801*

△*NIH Center for Macromolecular Modeling and Bioinformatics, University of Illinois at Urbana-Champaign, Urbana, IL, 61801*

E-mail: diwakar@illinois.edu

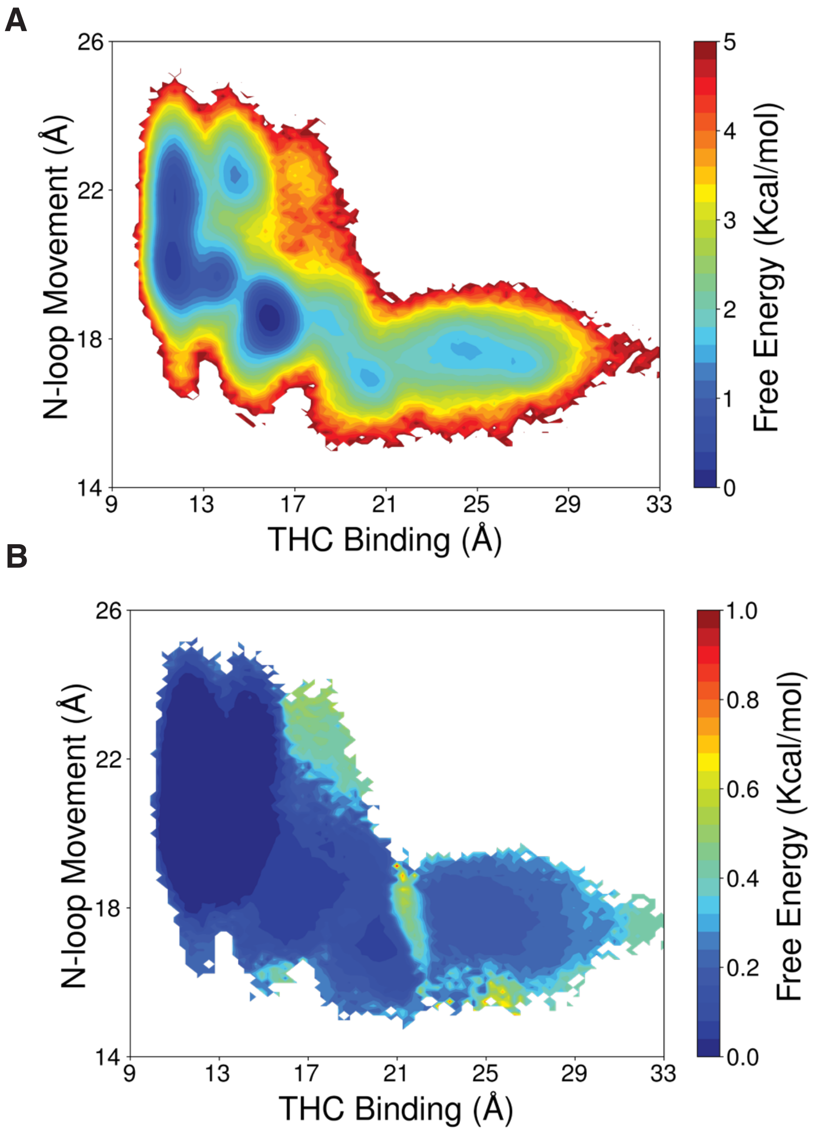

Figure S1: (A) Unweighted free energy landscape to capture THC binding and N-loop upward motion. (B) Errors on the free energy landscape projected on the same matrices. THC binding distance is measured between THC-C1' and TYR275$^{5.39}$-Cα (TM5) and N-loop upward motion is measured between MET103$^{\mathrm{N-loop}}$-Cα (N-loop) and ASP163$^{2.50}$-Cα (TM2). Errors are calculated with 500 rounds of bootstrap sampling with 80% of total number of trajectories (Method section).

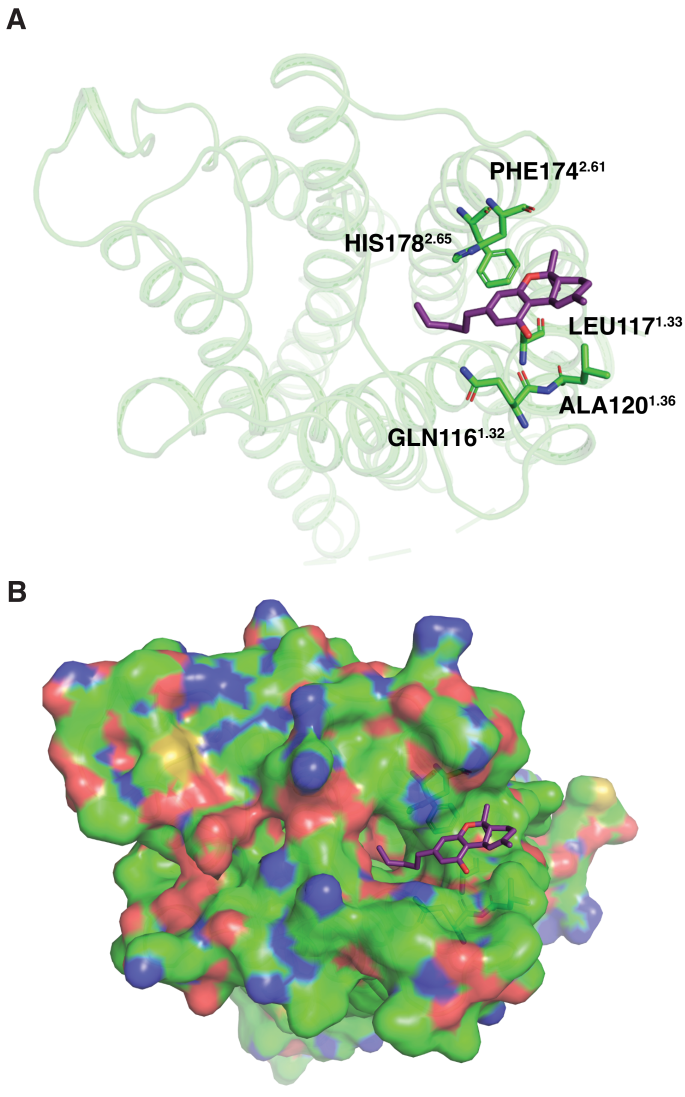

Figure S2: MD snapshot representing binding of THC from the extracellular region through the space between N-loop, TM1 and TM2 (top view). Proteins are represented as cartoon in (A) and as surface in (B). THC (color:violet) and interacting residues (color: green) are shown as stick representation.

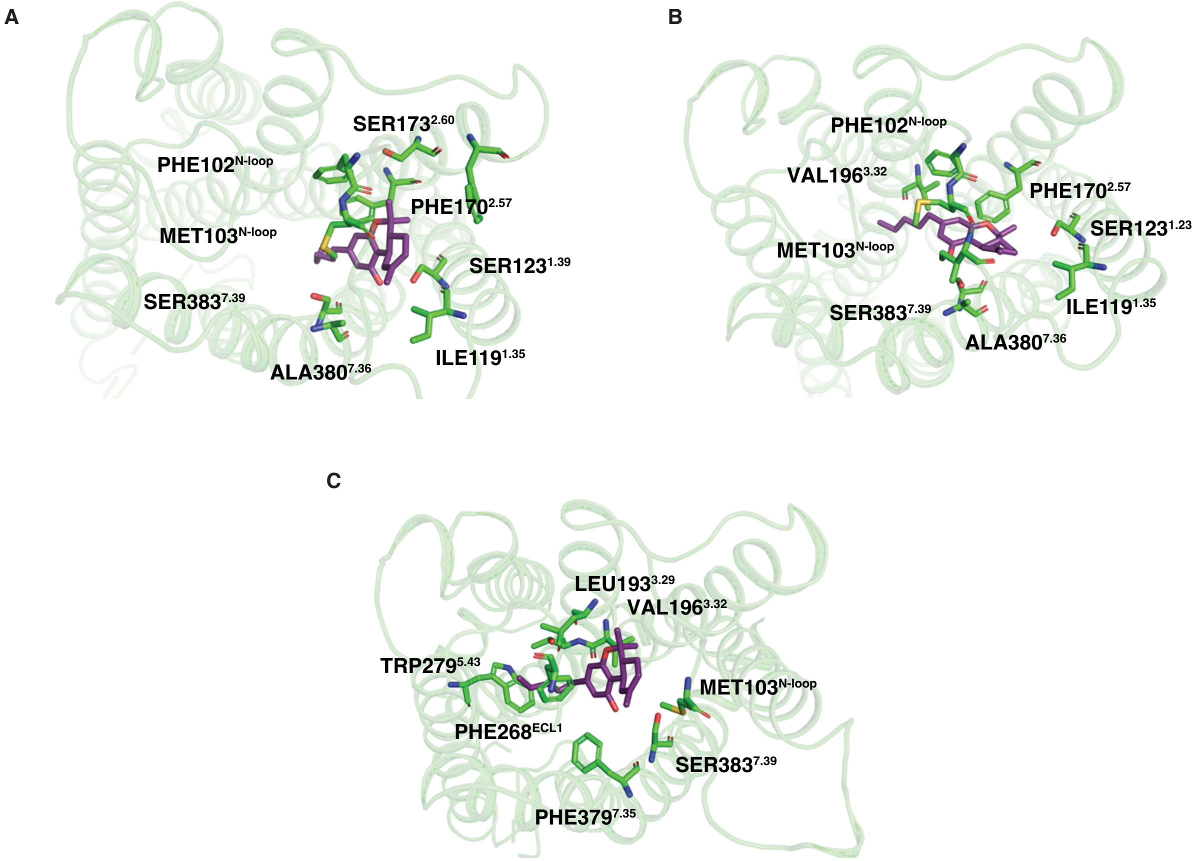

Figure S3: Important interactions between protein residues and THC at different stabilized positions during binding (top view). (A), (B) shows a representative structure from antagonist-like pose 1 and 2, respectively. (C) shows a representative structure from agonist-like pose. Stable interactions were measured using GetContacts package. Protein structures are shown as cartoon representation (color: green). THC (color: violet) and interactive residues (color: green) are shown as stick.

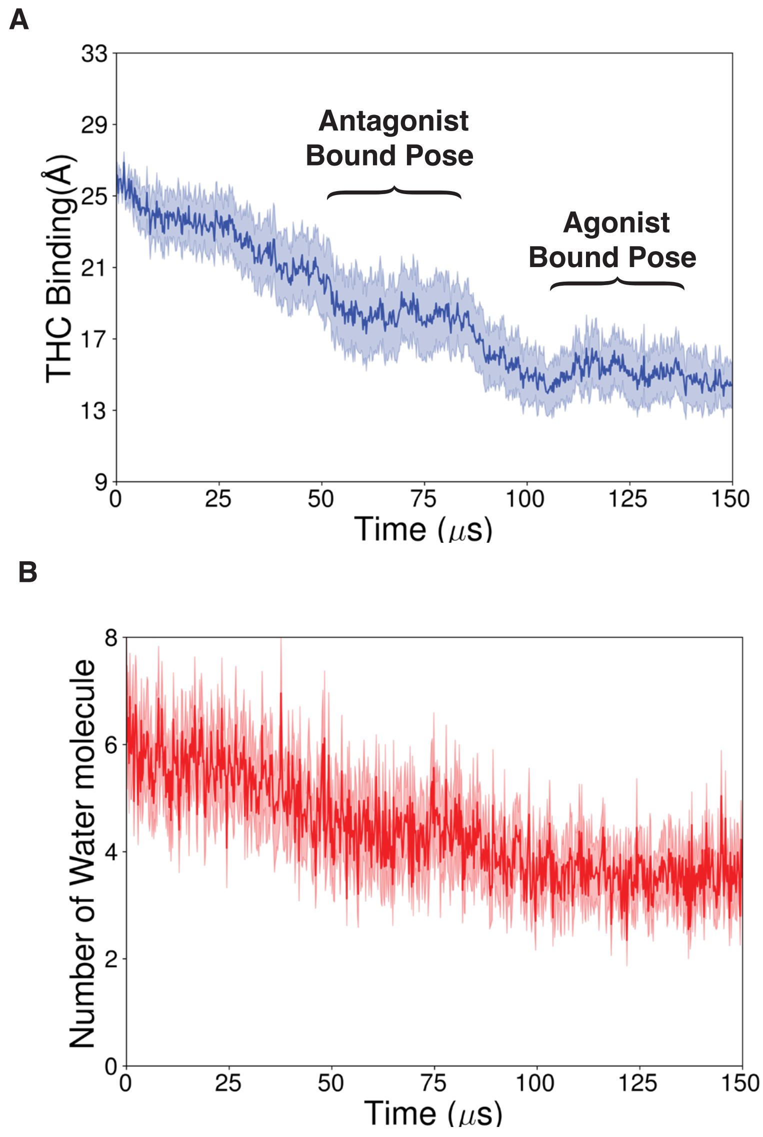

Figure S4: Kinetic Monte Carlo trajectory of 150 μs generated with the THC outside of the receptor. THC-C1' and TYR275$^{5.39}$-Cα (TM5) distance (A) and number of water molecules surrounding the the ligand (B) plotted against the time to show the transition during binding. Error bars are shown as semitransparent region. Error bars in the plot are calculated by running kMC simulations for 20 times.

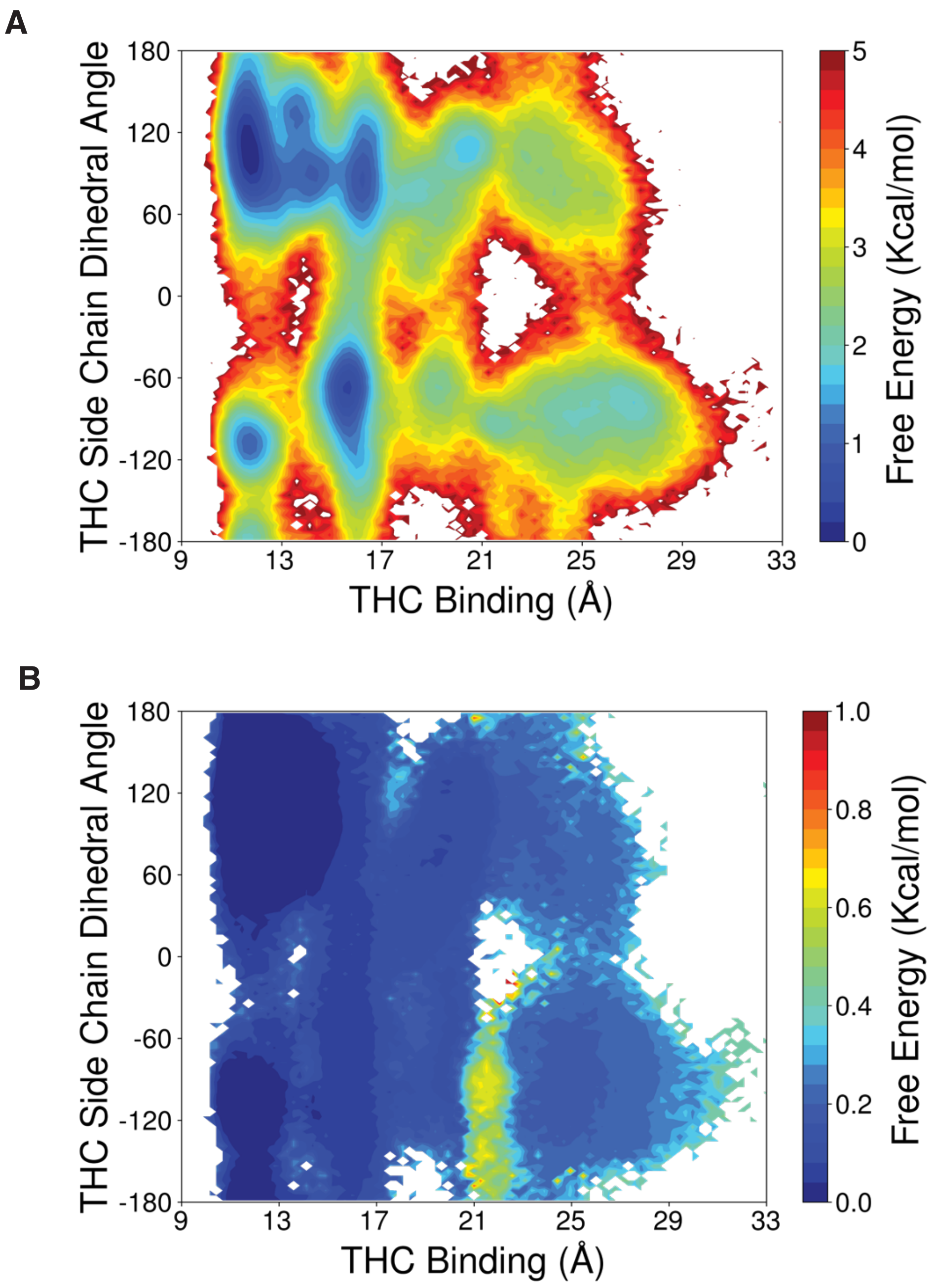

Figure S5: (A) Unweighted free energy landscape to capture THC binding and THC side chain dihedral. (B) Errors on the free energy landscape projected on the same matrices. THC binding distance is measured between THC-C1' and TYR275$^{5.39}$-Cα (TM5) and THC sidechain dihedral is measured between C2,C3,C1',C2'. Errors are calculated with 500 rounds of bootstrap sampling with 80% of total number of trajectories (Method section).

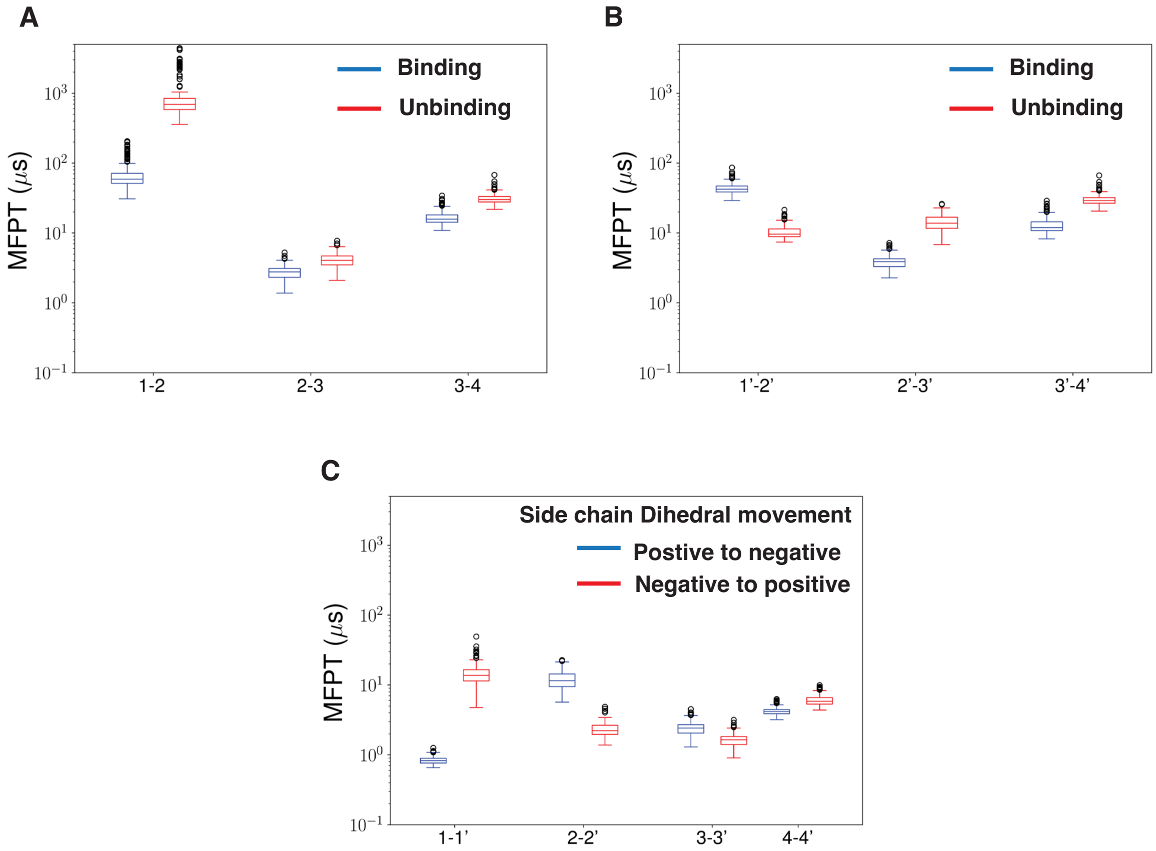

Figure S6: Box plot for mean free passage time between the macrostates as shown in Figure 4A. (A), (B) are showing the transitions between macrostates in top and bottom panel, respectively. Blue boxes are representing binding and red boxes are representing unbinding. (C) is representing MFPTs for dihedral transitions between macrostates with positive and negative dihedral.

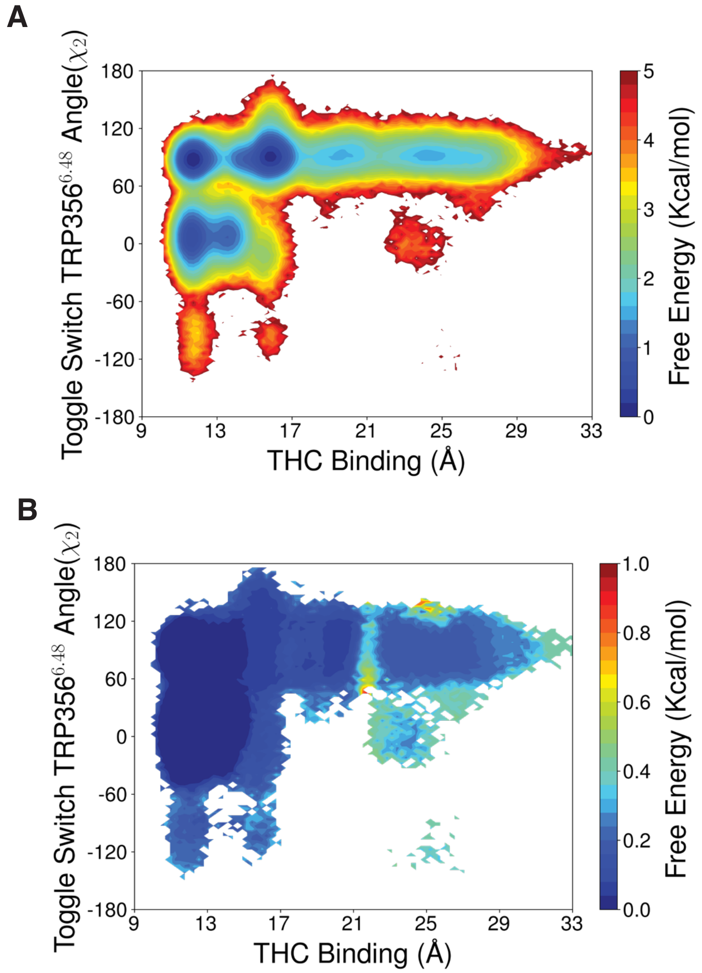

Figure S7: (A) Unweighted free energy landscape to capture THC binding and toggle switch TRP356 (helix 6) χ_2 angle. (B) Errors on the free energy landscape projected on the same matrices. THC binding distance is measured between THC-C1' and TYR275$^{5.39}$-Cα (TM5). Errors are calculated with 500 rounds of bootstrap sampling with 80% of total number of trajectories (Method section).

A

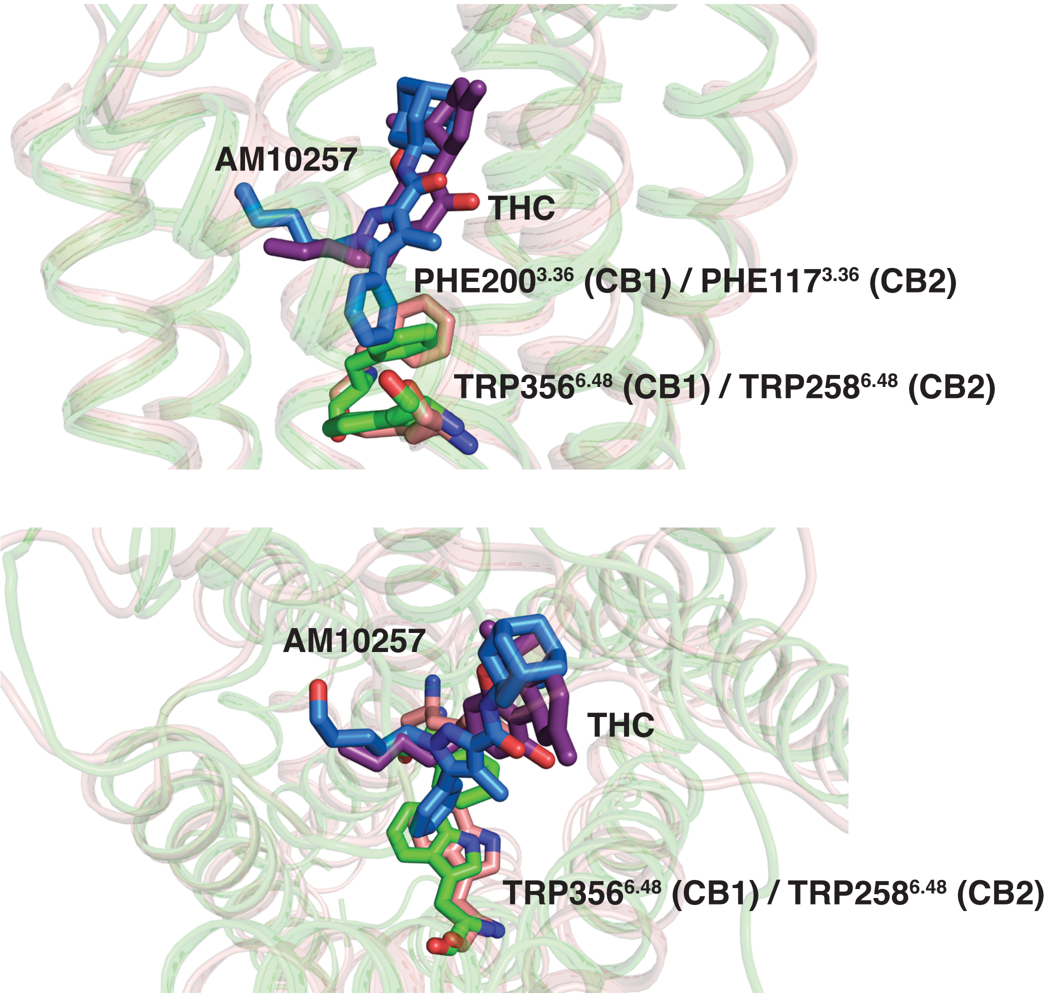

Figure S8: Comparison of partially active (state 1) (color: green) structure with inactive CB_2 (color: light brown) from side (A) and top view (B). Toggle switch residues and ligands (AM10257: blue,THC: violet) are shown as stick representation.

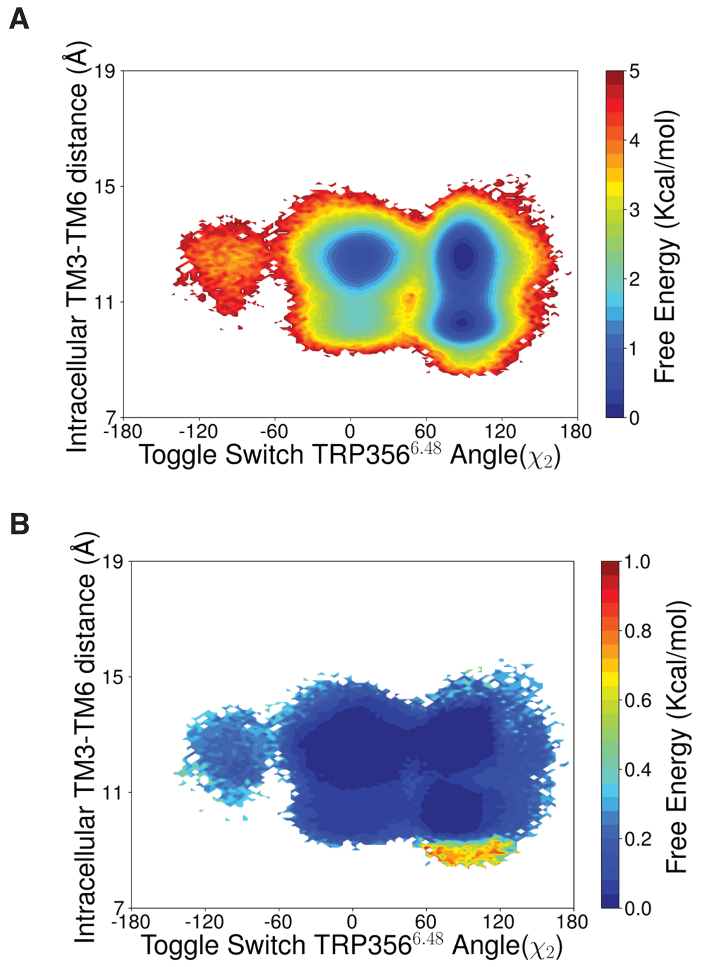

Figure S9: (A) Unweighted free energy landscape to capture toggle switch TRP356 (helix 6) χ_2 angle and intracellular TM6 movement of CB1. (B) Errors on the free energy landscape projected on the same matrices. Intracellular TM6 movement is measured ARG214$^{3.50}$-Cα (TM3) and LYS343$^{6.35}$-Cα (TM6). Errors are calculated with 500 rounds of bootstrap sampling with 80% of total number of trajectories (Method section).

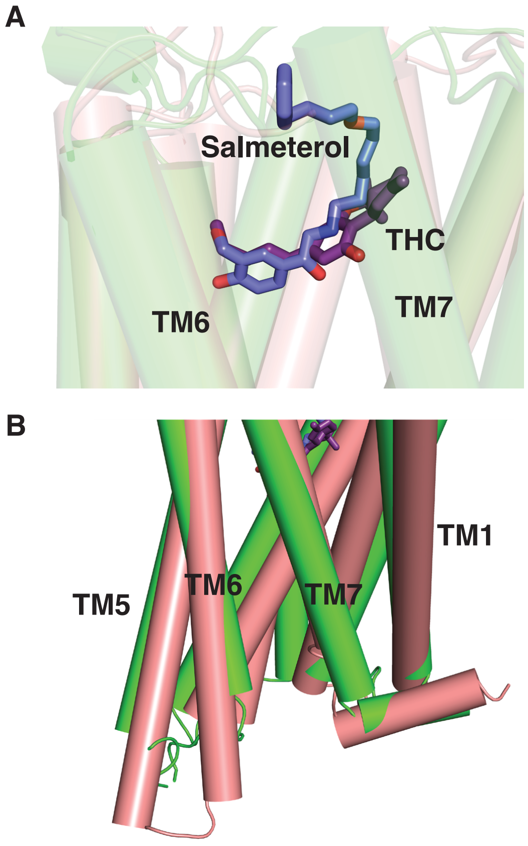

Figure S10: Comparison of partially active CB_1 (state 1) (color: green) structure with partially active β_2-AR (PDB ID: 6CSY, color: light brown) from side view. (A) focuses on the comparison of ligand bound pose of THC (color: violet) and Salmeterol (color: blue). (B) shows the TM orientation of both the receptor. ligands (salmeterol: blue, THC: violet) are shown as sticks.

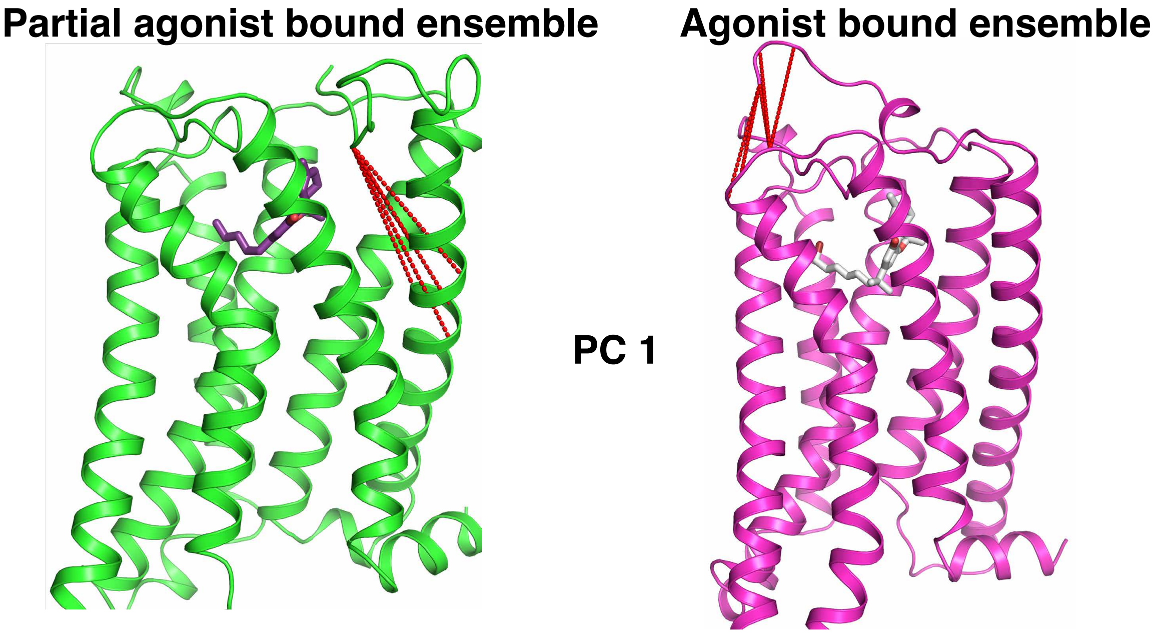

Figure S11: Five inter residue distances with highest weight contributions for principal component 1 (PC 1) for partial agonist (color: green) and agonist bound (color: pink) ensembles.

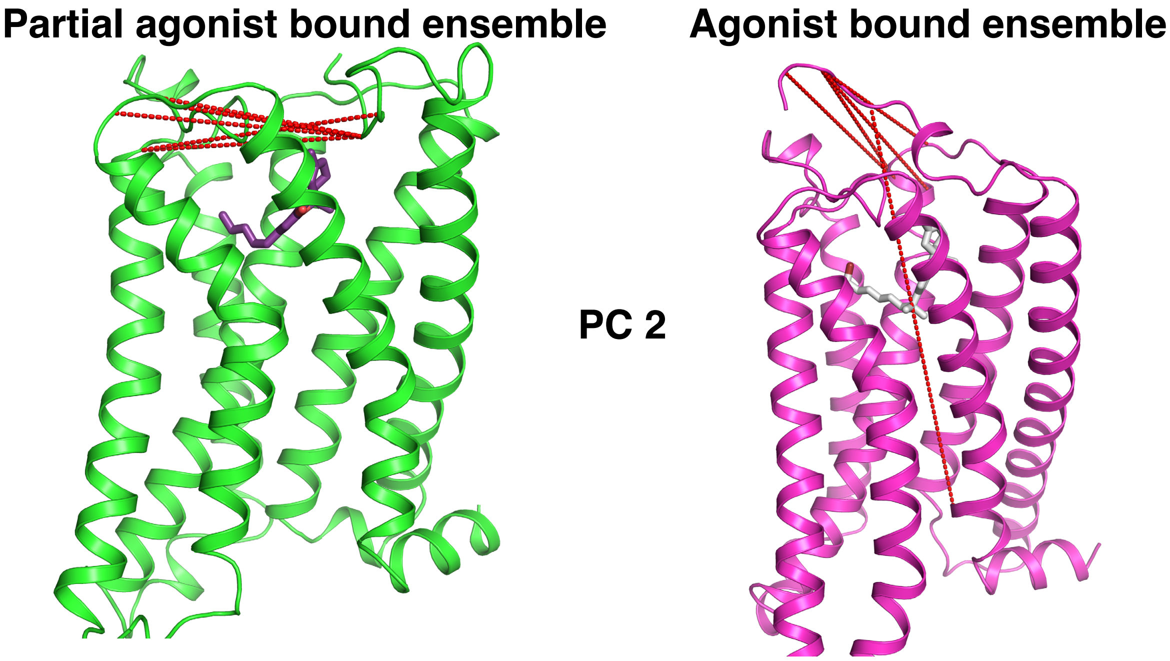

Figure S12: Five inter residue distances with highest weight contributions for principal component 2 (PC 2) for partial agonist (color: green) and agonist bound (color: pink) ensembles.

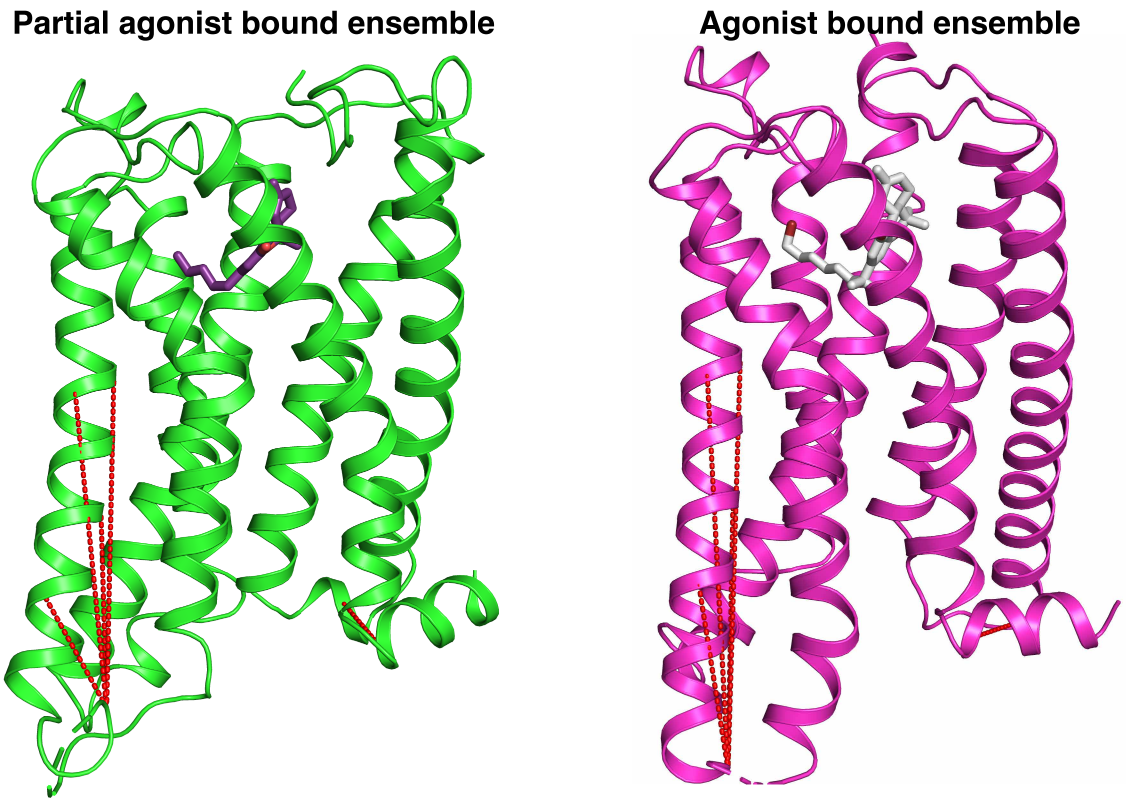

Figure S13: Five inter residue distances with highest K-L divergence between partial agonist (color: green) and agonist bound (color: pink) ensembles.

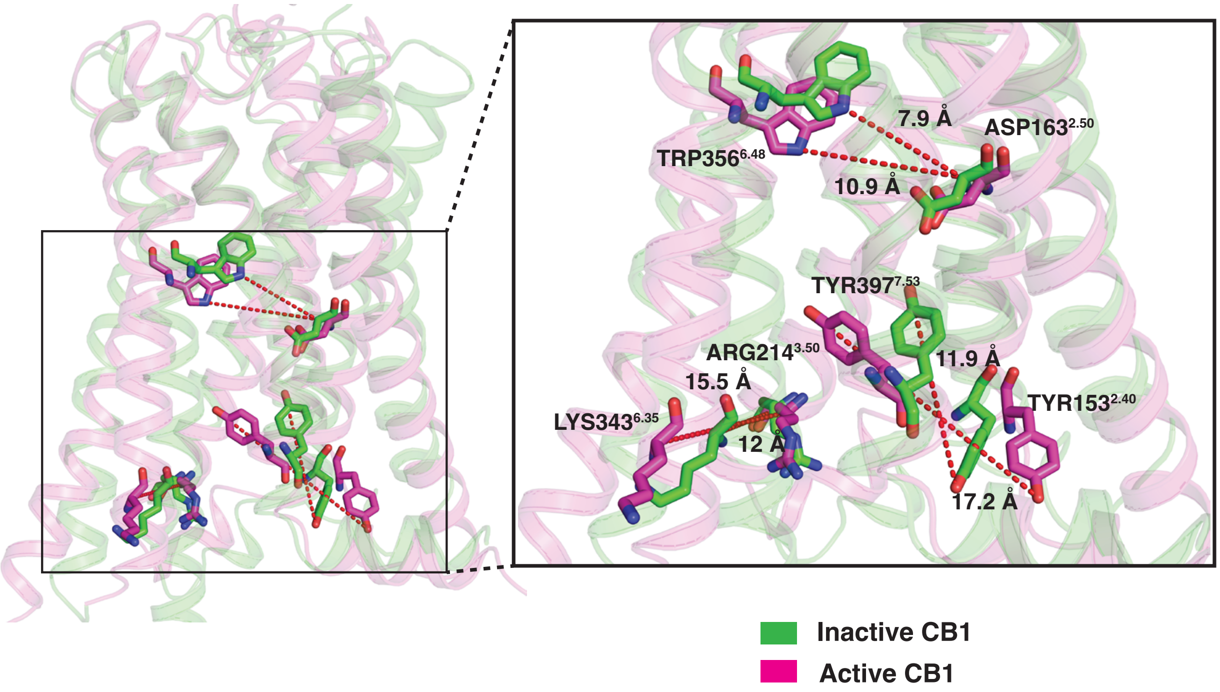

Figure S14: Comparison of activation microswitch conformations for active (PDB ID: 5XRA; color: Pink) and inactive (PDB ID: 5TGZ; color: Green) structure of CB1. Distances that are measured to represent these conformational differences are shown in the figure.

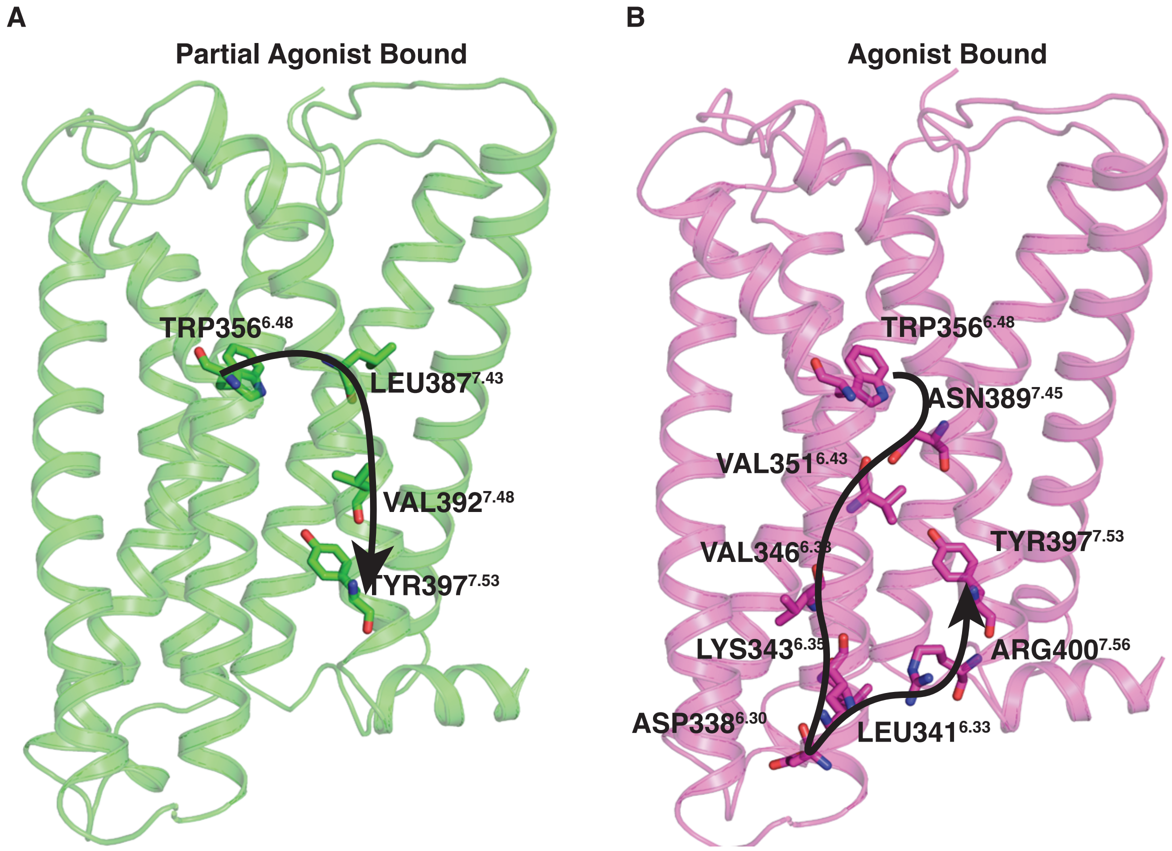

Figure S15: Shortest allosteric pathway between toggle switch and NPxxY region calculated using the residue dihedral mutual information for partial agonist bound ensemble and agonist bound ensemble.

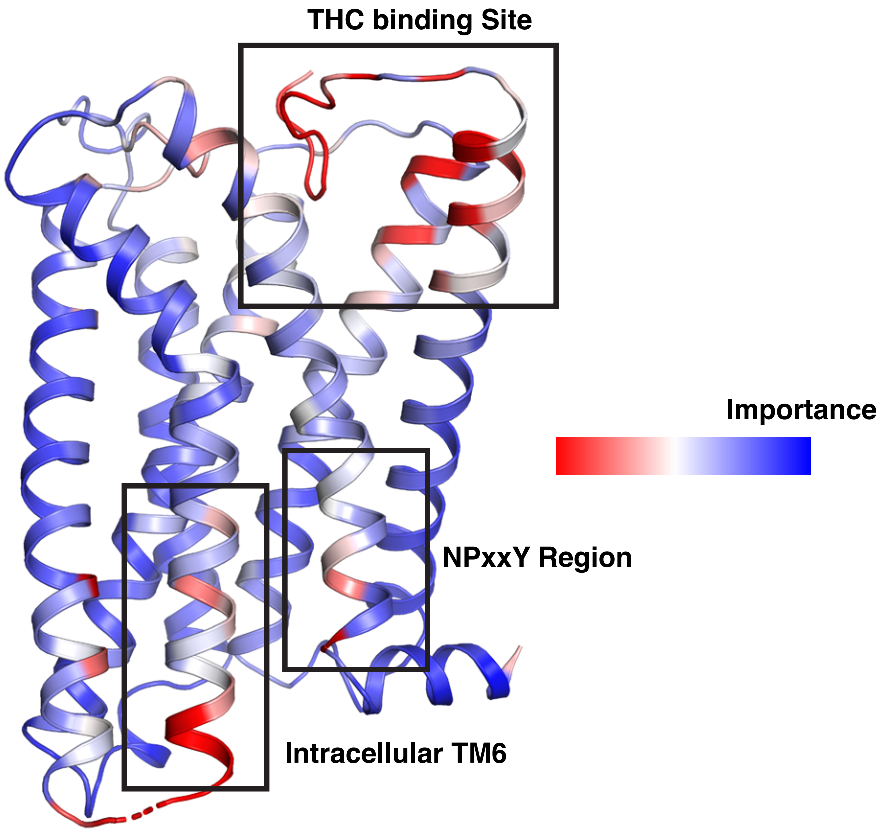

Figure S16: Important residue movement during the THC binding extracted using PCA. PCA analysis was performed on 20000 frames selected from entire binding ensemble based on the cluster probability calculated using MSM.

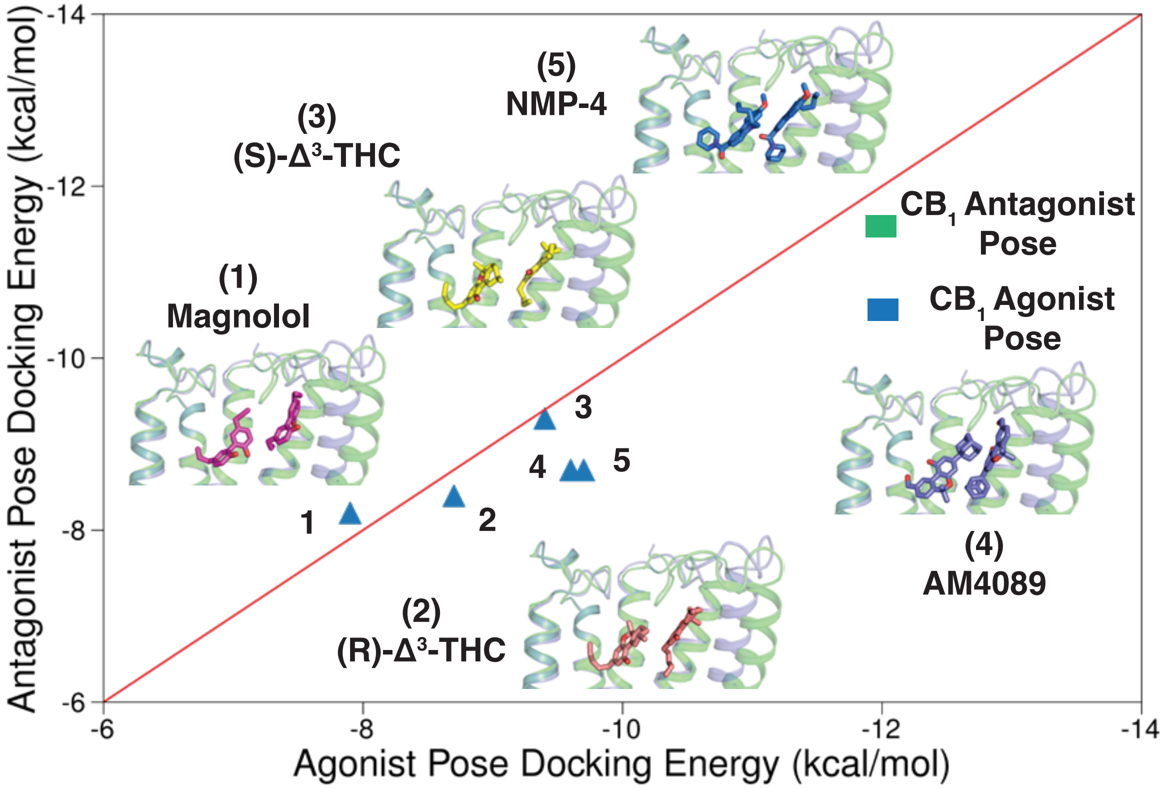

Figure S17: Agonist pose docking energy is plotted against antagonist pose docking energy for five different partial agonists. Protein with docked partial agonists in antagonist pocket(color: green) and agonist pocket (color: purple) are superimposed on each other. Five partial agonists are represented as stick with different color (Magnolol:pink,(R)-Δ^3-THC: light brown,(S)-Δ^3-THC: yellow, AM4089: purple, NMP-4: blue).

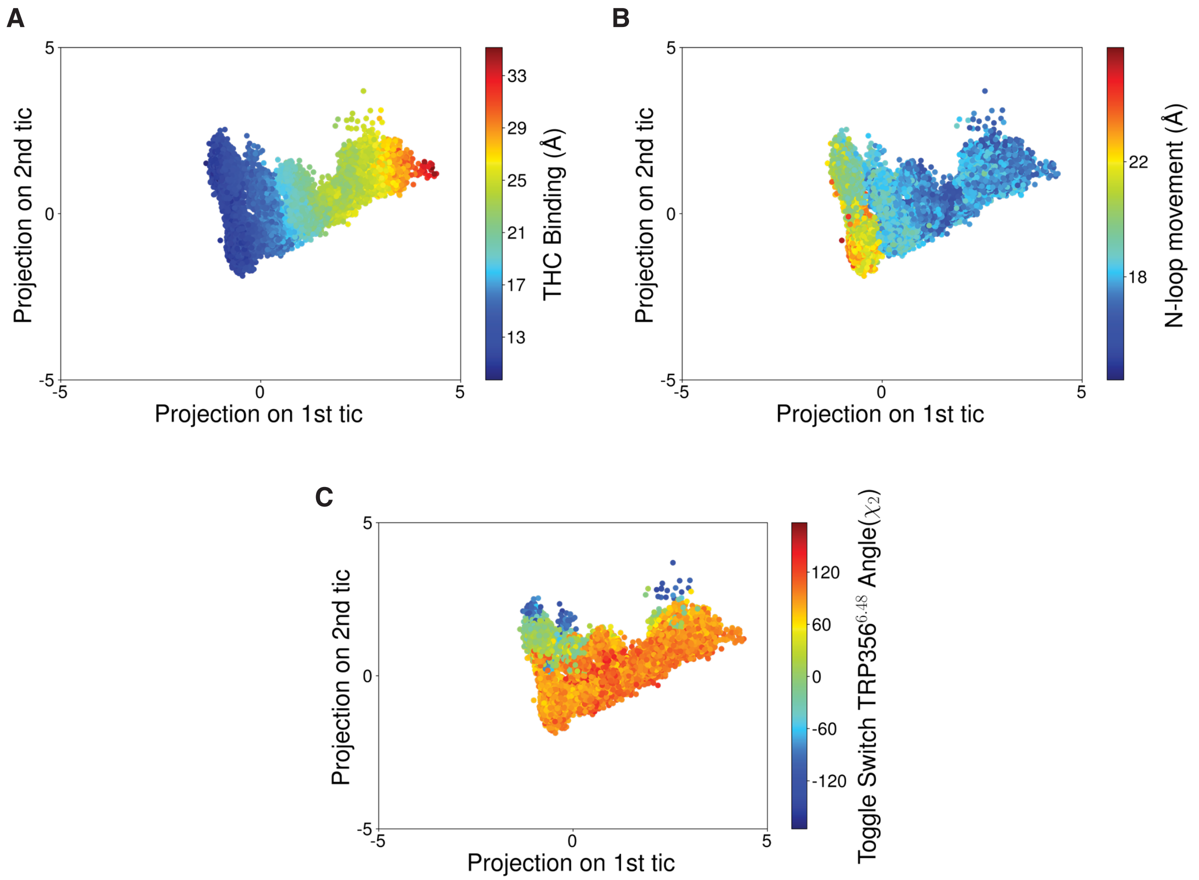

Figure S18: Correlation of 1st and 2nd tic components with system features. First tic component is highly correlated with THC binding (A) and N-loop movement (B). Second tic component is highly correlated with TRP356$^{6.48}$ χ_2

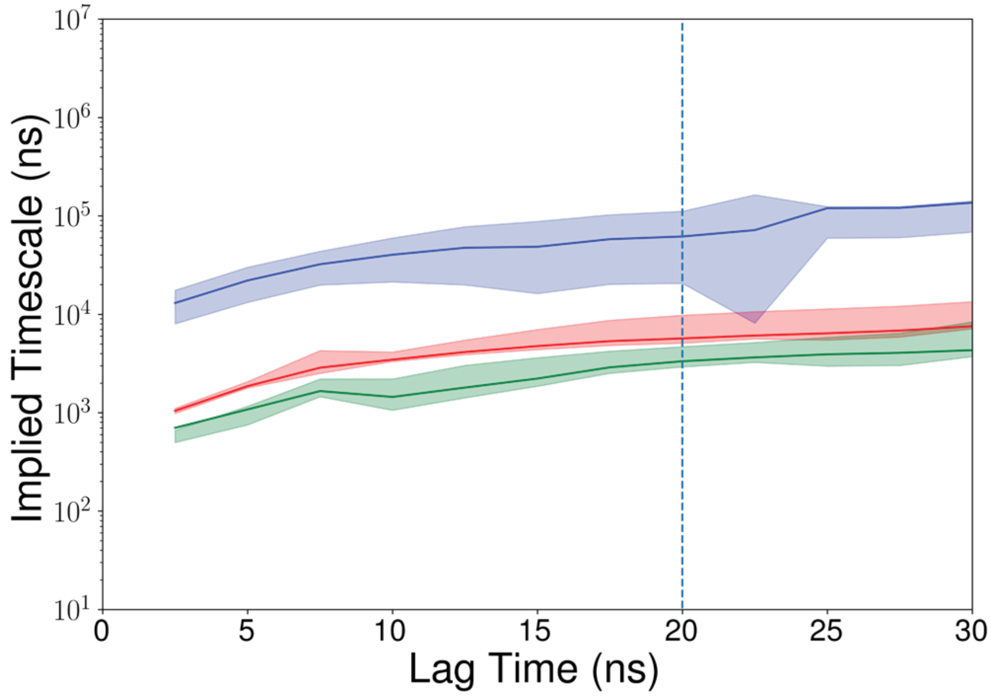

Figure S19: Logarithmic convergence of timescales (2nd, 3rd and 4th highest eigenvalues) plotted against various lag times. Lag time of 20 ns used to build our MSM. Errors are calculated with 10 rounds of bootstrap sampling with 80% of total number of trajectories (Method section).

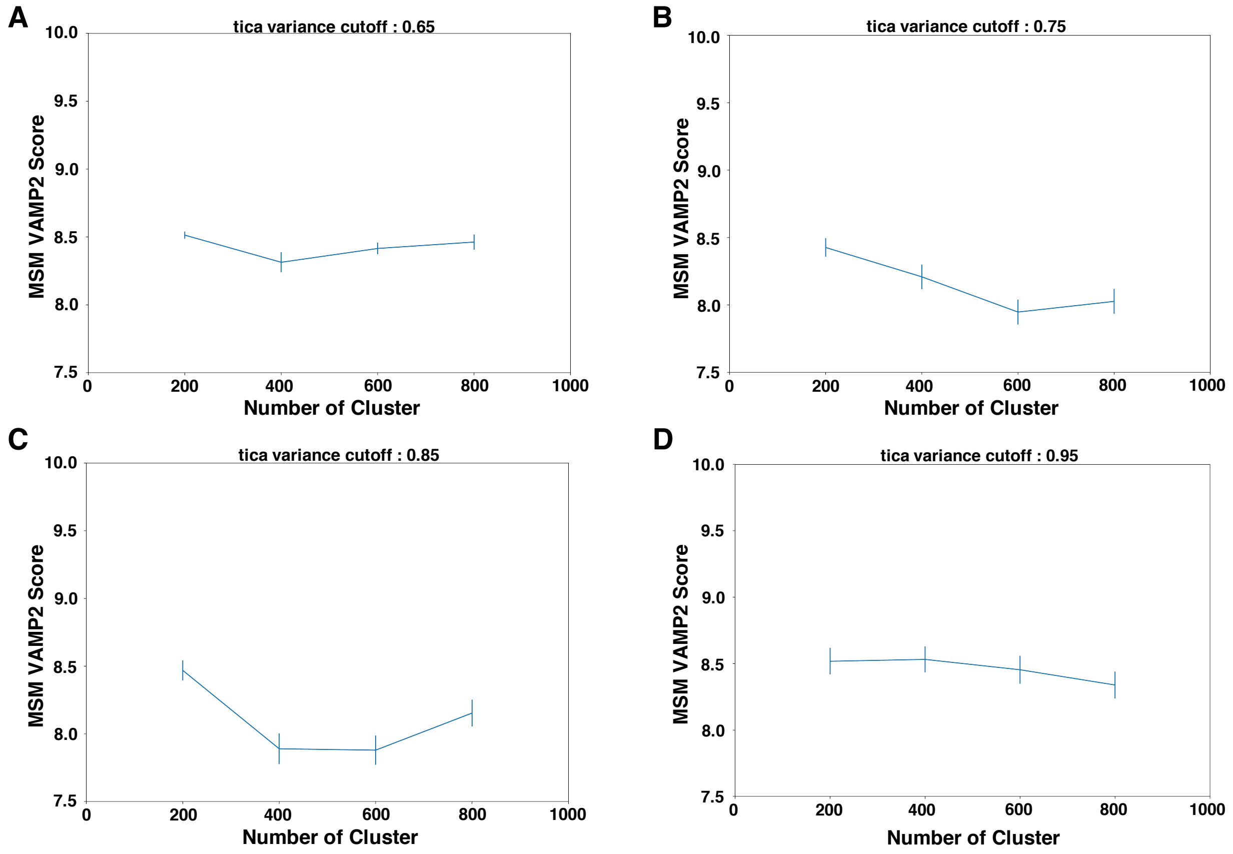

Figure S20: Optimal cluster number are chosen based on VAMP2 score of MSM based on different tic variance cutoff. MSM with tic variance of 95% and 200 clusters were selected for our simulation.

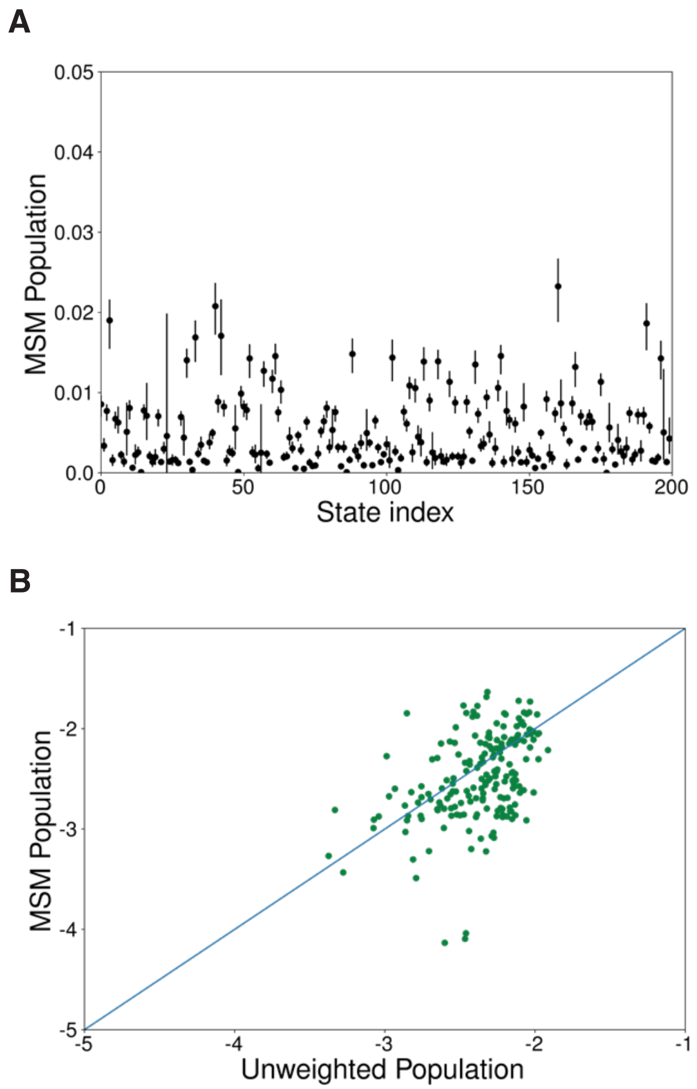

Figure S21: (A) Probability density of each MSM state. (B) MSM population vs Raw count in each clustered state. Errors are calculated with 10 rounds of bootstrap sampling with 80% of total number of trajectories (Method section).

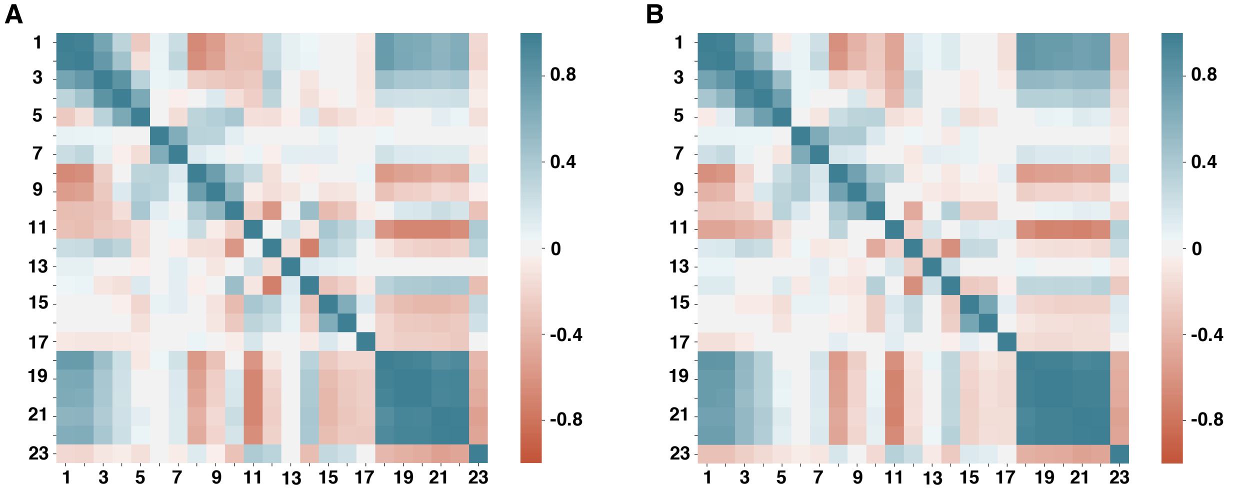

Figure S22: (A) Unweighted and (B) MSM weighted co-relation matrix of the features used to build MSM. Features corresponding feature number can be found from Table 1.

Table S1: Distance and angle features used to build Markov state model.

Position	Number	Type	Feature
Extracellular helical movement	1	distance	ASN116^{TM1}(CA)-PHE189^{TM3}(CA)
	2	distance	ASN116^{TM1}(CA)-PRO251^{TM4}(CA)
	3	distance	ASN116^{TM1}(CA)-LEU276^{TM5}(CA)
	4	distance	ASN116^{TM1}(CA)-ASP366^{TM6}(CA)
	5	distance	ASN116^{TM1}(CA)-THR377^{TM7}(CA)
	6	distance	ASP176^{TM2}(CA)-PHE189^{TM3}(CA)
	7	distance	ASP176^{TM2}(CA)-PRO251^{TM4}(CA)
	8	distance	ASP176^{TM2}(CA)-LEU276^{TM5}(CA)
	9	distance	ASP176^{TM2}(CA)-ASP366^{TM6}(CA)
	10	distance	ASP176^{TM2}(CA)-THR377^{TM7}(CA)
N-loop movement	11	distance	MET103^{N-loop}(CA)-ASP163^{TM2}(CA)
	12	distance	MET103^{N-loop}(CA)-PHE268^{ECL2}(CA)
Toggle switch movement	13	Dihedral Angle (χ_2)	PHE200^{TM2}
	14	Dihedral Angle (χ_2)	TRP356^{TM6}
Intracellular helical movement	15	distance	PHE155^{TM2}-LYS343^{TM6}
	16	distance	ARG214^{TM3}-LYS343^{TM6}
	17	distance	TYR153^{TM2}-TYR397^{TM7}
THC binding	18	distance	THC(C3)-PHE268^{ECL2}
	19	distance	THC(C1')-TYR275^{TM5}
	20	distance	THC(C1')-TRP279^{TM5}
	21	distance	THC(C5')-TYR275^{TM5}
	22	distance	THC(C5')-TRP279^{TM5}
	23	Dihedral Angle (C2-C3-C1'-C2')	THC

Table S2: Amount of simulation (μs) in each round of adaptive sampling

Sampling Round	Total time
1	27.83
2	4.83
3	4.68
4	9.18
5	5.78
6	4.13
7	11.93
8	11.49
9	11.44
10	5.13
11	4.93
12	4.73
13	2.11
14	6.68
15	5.37
16	2.57
17	2.73
18	7.98
19	9.49
Total time	143.01
